# Comparative Genomic Analysis Indicates that Niche Adaptation of Terrestrial *Flavobacteria* Is Strongly Linked to Plant Glycan Metabolism

**DOI:** 10.1371/journal.pone.0076704

**Published:** 2013-09-26

**Authors:** Max Kolton, Noa Sela, Yigal Elad, Eddie Cytryn

**Affiliations:** 1 Institute of Soil, Water and Environmental Sciences, the Volcani Center, Agricultural Research Organization, Bet Dagan, Israel; 2 Department of Plant Pathology and Weed Research, the Volcani Center, Agricultural Research Organization, Bet Dagan, Israel; 3 Institute of Plant Sciences and Genetics in Agriculture, The Robert H. Smith Faculty of Agriculture, Food and Environment, the Hebrew University of Jerusalem, Rehovot, Israel; University of Mississippi Medical Center, United States of America

## Abstract

*Flavobacteria* are important members of aquatic and terrestrial bacterial communities, displaying extreme variations in lifestyle, geographical distribution and genome size. They are ubiquitous in soil, but are often strongly enriched in the rhizosphere and phyllosphere of plants. In this study, we compared the genome of a root-associated *Flavobacterium* that we recently isolated, physiologically characterized and sequenced, to 14 additional *Flavobacterium* genomes, in order to pinpoint characteristics associated with its high abundance in the rhizosphere. Interestingly, flavobacterial genomes vary in size by approximately two-fold, with terrestrial isolates having predominantly larger genomes than those from aquatic environments. Comparative functional gene analysis revealed that terrestrial and aquatic Flavobacteria generally segregated into two distinct clades. Members of the aquatic clade had a higher ratio of peptide and protein utilization genes, whereas members of the terrestrial clade were characterized by a significantly higher abundance and diversity of genes involved in metabolism of carbohydrates such as xylose, arabinose and pectin. Interestingly, genes encoding glycoside hydrolase (GH) families GH78 and GH106, responsible for rhamnogalacturonan utilization (exclusively associated with terrestrial plant hemicelluloses), were only present in terrestrial clade genomes, suggesting adaptation of the terrestrial strains to plant-related carbohydrate metabolism. The Peptidase/GH ratio of aquatic clade Flavobacteria was significantly higher than that of terrestrial strains (1.7±0.7 and 9.7±4.7, respectively), supporting the concept that this relation can be used to infer *Flavobacterium* lifestyles. Collectively, our research suggests that terrestrial *Flavobacteria* are highly adapted to plant carbohydrate metabolism, which appears to be a key to their profusion in plant environments.

## Introduction

The Gram negative genus *Flavobacterium* (phylum *Bacteriodetes*) encompasses over 100 different strains that are characterized by exceptional environmental niche heterogeneity ranging from Arctic lakes to thermal springs [1]. They are important members of the bacterial community in both aquatic and terrestrial environments, where their relative abundance can in some cases reach up to 20% [2-8]. Several *Flavobacterium* species are the causative agent of severe fish diseases [9,10], while others are associated with plant protection and growth promotion [11,12] and with bioremediation in soils and marine sediments [13,14].

A myriad of recent studies strongly suggest that flavobacterial abundance is significantly enhanced in the rhizosphere and phyllosphere of terrestrial plants [2,3,7]. For example, Bulgarelli et al., (2012) showed that the roots of *A. thaliana*, grown in different natural soils under controlled environmental conditions, are preferentially colonized by *Flavobacteria*, where the relative abundance on roots (5 to 15% of total defined families) was between 20 to 100-fold higher than in bulk and rhizosphere soil. This was also supported by a recent study by Bodenhausen et al., (2013) that determined that *Flavobacterium* were among the most dominant genera on both roots and leaves (4 to 10%) of wild *A. thaliana*.

Adaptation of *Flavobacterium* to such a wide array of heterogeneous environments is undoubtedly associated with genetic plasticity. Thus, in order to identify functional mechanisms associated with the enhanced rhizosphere and phyllosphere competence of *Flavobacterium* , we conducted comprehensive comparative genomic analysis of a model root-associated *Flavobacterium* strain that we recently sequenced [15] and fourteen additional *Flavobacterium* genomes isolated from plant roots, phyllosphere, soil, marine and freshwater ecosystems and fish tissue. Although some genes were ubiquitous to all flavobacteria, a myriad of functional genes were unique to terrestrial environments. These included a diverse group of genes encoding plant polysaccharide degrading enzymes, which appear to be central to plant-associated flavobacterial metabolism.

## Materials and Methods

### *Flavobacteria* isolation and characterization

Mature bell pepper (*Capsicum annuum* L. cv. Maccabi) plants were grown in potting mixtures in an experimental pest- and disease-free greenhouse as previously described [6,16,17]. Plant roots were washed briefly to remove lightly adhering soil and homogenized in sterile saline (0.85% NaCl), and homogenate 10-fold serial dilutions were plated on Casitone-Yeast Extract medium (CYE) [18]. Isolated *Flavobacterium* spp. were tested for gliding motility, presence of flexirubin, hydrolytic activities: protease, lipase, chitinase, amylase, cellulase, β-galactosidase, nannanase, xylanase, urease, and expression of plant growth promotion and protection features: ACC-deaminase, NH_3_ and hydrogen cyanide (HCN) production, using previously described standard procedures [19-24]. One of the isolates, *Flavobacterium* sp. F52, selected based on its enhanced biocontrol capacity, was sequenced and used for further analyses as described below.

### Analyzed strains

Comparative analysis was performed on 15 publically-available flavobacterial genomes from both aquatic and terrestrial environments including *Flavobacterium* sp. F52. Their names and GenBank accession numbers (http://www.ncbi.nlm.nih.gov/Genbank) are presented in Table 1. Additionally, the *Bacteroidetes* species Porphyromonas *gingivalis* W83 (NC002950.2) was used as an out-group genome for phylogenetic and metabolic potential analyses.

**Table 1:** *Flavobacterium* spp. analyzed in this study.

Strain*	Genome size (Mbp)	GC content (%)	GenBank accession number	Isolation site	References
*Flavobacterium* *johnsoniae* UW101 (F)	6.1	34	NC009441	Soil; UK	[63]
*Flavobacterium* sp. F52 (D)	5.34	34	NZAKZQ00000000	Rhizosphere of pepper; Israel	[15]
*Flavobacterium* sp. URHB0058 (D)	5.26	34	AUEU00000000	Forest soil; North America	DOE Joint Genome Institute
*Flavobacterium* sp. WG21 (D)	5.19	36	NZMYW00000000	Wintergreen Lake; USA	University of Notre Dame
*Flavobacterium* sp. CF136 (D)	5.12	34	NZAKJZ00000000	Tree rhizosphere; USA	[64]
*Flavobacterium* *rivuli* DSM 21788 (D)	4.49	40	NZKB899988	Hard water stream; Germany	DOE Joint Genome Institute
*Flavobacterium* sp. B17 (D)	4.17	37	BACY00000000	Rice shoot; Japan	NCBI
*Flavobacterium* *soli* DSM 19725 (D)	4.0	36	AUGO00000000	Soil samples; South Korea	DOE Joint Genome Institute
*Flavobacterium* sp. ACAM 123 (D)	3.96	35	NZAJXL01000285	Burton Lake; Antarctic lake	NCBI
*Flavobacterium* *frigoris* PS1 (D)	3.93	34	NZAHKF00000000	Sea ice, diatom layer; Antarctic lake	[65]
*Flavobacterium* *branchiophilum* FL-15 (F)	3.56	33	NC016001	Diseased sheatfish; Hungary	[66]
*Flavobacterium* *columnare* ATCC 49512 (F)	3.16	31	NC016510	Skin lesion of trout fry; France	[67]
*Flavobacterium* *antarcticum* DSM 19726 (D)	3.08	35	NR042998	Soil sample; Antarctica	DOE Joint Genome Institute
*Flavobacterium* *indicum* GPTSA100-9 (F)	2.99	31.5	NC017025	Warm spring water; India	[68]
*Flavobacterium* *psychrophilum* JIP02/86 (F)	2.86	33	NC009613	Kidney of rainbow trout; France	[69]

(*) F – full completed genome; D – draft genome.

### Phylogenetic analyses

Phylogenetic relationships among all sequenced *Flavobacterium* species were investigated based on 16S rRNA gene sequences and concatenated multilocus sequence analysis (MLSA) of 10 highly conserved housekeeping genes: *serS*, *aroE*, *atpD*, *dnaE*, *guaA*, *gyrB*, *mutL*, *pyrC*, *recA*, and *rpoB*, as previously described [25,26]. Additionally, we applied an e-value-based clustering algorithm from reciprocal all-by-all BLASTP analysis of all coding sequences of the investigated genomes using the Hal automated pipeline [27]. Phylogenetic trees illustrating the relationships of 227 common concatenated core genes from the 15 flavobacterial genomes and the outlier *P. gingivalis* W83 were constructed with the Phylogenetic Maximum Likelihood (PhyML) method, using the PhyML 3.0 algorithm with 100 bootstrap replicates and the LG matrix of amino-acid substitution model (http://www.atgc-montpellier.fr/phyml/;( [28,29])). Sequences of the 16S rRNA genes and concatenated housekeeping genes were aligned with MUSCLE [30]. To assess the robustness of the phylogenetic tree topology, bootstrap consensus trees were inferred from 1000 replicates. Maximum likelihood phylogenetic trees were generated and/or visualized by using MEGA 5.1 software with default parameters [31].

### *Flavobacterium* *core* and niche-specific genomes

Orthologs were defined by identifying unique pairwise reciprocal best hits, with at least 60% similarity in amino acid sequence and less than 30% difference in protein length. To define the flavobacterial core genome, we used the 15 available genomes described above. For niche-specific genome identification we used the completed genomes: *F* *. branchiophilum* FL-15; *F* *. psychrophilum* JIP02/86; *F* *. columnare* ATCC 49512 and *F* *. indicum* GPTSA100-9 as representatives of the aquatic clade; and the completed genome of *F* *. johnsoniae* UW101 with two draft genomes *Flavobacterium* sp. F52 and *Flavobacterium* sp. CF136 as representatives of the terrestrial clade. To determine the functional roles of the core and niche-specific genes, open reading frames (ORFs) were assigned to gene onthology (GO) groups using Blast2GO software, and tested for enrichment using Fisher’s exact test [32]. Only statistically different GO’s were included in future analyses. These GO’s were manually grouped to metabolic pathways and presented as a ratio between inspected set proteins to the reference set. Thus a ratio above or below one indicates over or under representation, respectively of specific group in an inspected core genome.

### Whole-genome functional gene comparison

The fifteen analyzed *Flavobacterium* genomes were annotated and distributed to SEED categories and subsystems using the RAST server (http://RAST.nmpdr.org [33]). The numbers of annotated and distributed genes across SEED categories and subsystems were normalized to the total number of genes in an individual genome. A Bray-Curtis distance matrix encompassing the relative abundances of each defined SEED categories or subsystem in the fifteen genomes was used for cluster analysis in order to assess the functional gene similarity of the flavobacterial strains. Statistically significant differences in allocation of genome resources to SEED categories and subsystems between bacterial clades was indicated by Student T-Test (*P*<0.05) using the SPSS 18 software package (SPSS inc., Chicago, Illinois). Bray-Curtis distance matrix calculation and cluster analysis with 1000 bootstrap replicates was performed by using the PAleo-ecology STatistics freeware (PAST) package, version 2.03 (http://folk.uio.no/ohammer/past/ [34]), and the representation of the generated clusters was performed with MEGA 5.1 software [31].

### Prediction of carbohydrate and peptide utilization

Sequence similarity based annotation was performed to identify genes with functional motifs encoding carbohydrate-binding and metabolic enzymes. Predictions were automatically determined using the CAZymes Analysis Toolkit (http://mothra.ornl.gov/cgi-bin/cat.cgi) applying the association rule-learning algorithm [35] and the dbCAN prediction web server (http://csbl.bmb.uga.edu/dbCAN/ [36]). Sequences with similar predictions based on both methods were selected and named according to CAZy classifications [37], and the abundance and diversity of specific carbohydrate-associated enzymes in the analyzed genomes was determined. Peptidase (protease) encoding genes in the flavobacterial genomes were predicted according to the MEROPS classification (http://merops.sanger.ac.uk/cgi-bin/blast [38]). Shannon diversity indices for CAZy classes were calculated with PAST version 2.03 [34] and statistical significant differences between bacterial clades was indicated by Student T-Test (*P*<0.05) using the SPSS 18 software package (SPSS inc., Chicago, Illinois).

## Results

### Isolation and characterization of root-associated *flavobacteria*

Twenty five flavobacterial strains were isolated from bell pepper roots as described above. Physiological analyses revealed that almost all of these isolates can successfully propagate at a wide range of temperatures (10-40°C) and pH values (4-9) (Table S1). Moreover characterization of enzymatic hydrolytic activities of this isolates reveled their high potential to utilize plant related polysaccharides such as mannan, xylan and cellulose (Table S1). In addition, all of the isolated root-associated flavobacteria produced ammonia and cyanide which are known players in plant-bacterial interaction (Table S1). Integrative analyses of these traits led us to select *Flavobacterium* sp. F52 as a model strain for studying *Flavobacterium* -root interactions.

### Genomic features of *Flavobacterium* *strains*

A summary of the features of each of the 15 flavobacteria genomes used for the comparative analyses conducted in this study is provided in Table 1. The flavobacteral genomes vary in size by approximately two-fold (ranging from 2.9 to 6.1 Mbp) with the number of gene coding sequences (CDSs) ranging from 2,432 to 5,017. The genomes of *Flavobacterium* strains isolated from temperate soil and rhizosphere environments were predominantly larger than those from aquatic ones, with the exception of *F* *. antarcticum* , *F* *. rivuli* and *Flavobacterium* sp. WG21 (Table 1, Figure S1).

### Phylogenetic analysis

**Figure 1:**
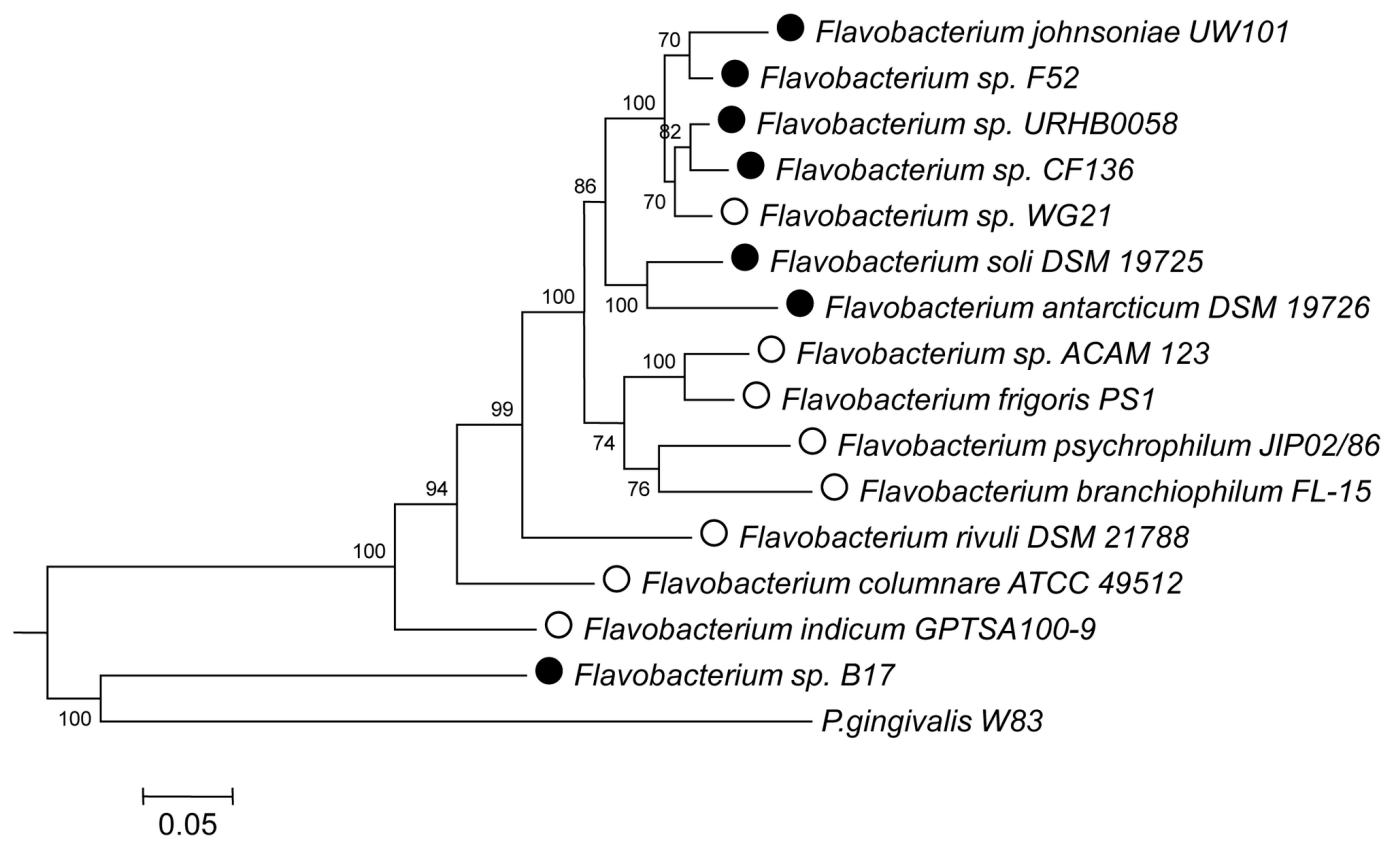
Phylogenetic relationships of MLSA genes in *Flavobacterium* strains. Maximum Likelihood analysis based on concatenated alignments of ten highly conserved housekeeping genes: *serS*, *aroE*, *atpD*, *dnaE*, guaA, *gyrB*, *mutL*, *pyrC*, *recA*, and *rpoB*. Bootstrap values are shown next to the branch nodes. Open and black circles represent aquatic and terrestrial isolates, respectively.

We estimated flavobacterial phylogenetic relationships by constructing maximum likelihood trees based on 16S rRNA genes (Figure S2), MLSA (Figure 1) and whole-genome comparisons of common proteins (Figure S3). MLSA and 16S rRNA gene-based analyses failed to show a clear-cut environmental-based distribution of flavobacteria (Figure 1; Figure S2), although MLSA-based phylogenetic analysis revealed that most of soil and plant-associated strains formed a strongly related paraphyletic sub-cluster (Figure 1).

### Functional characterization of flavobacterial isolates

**Figure 2:**
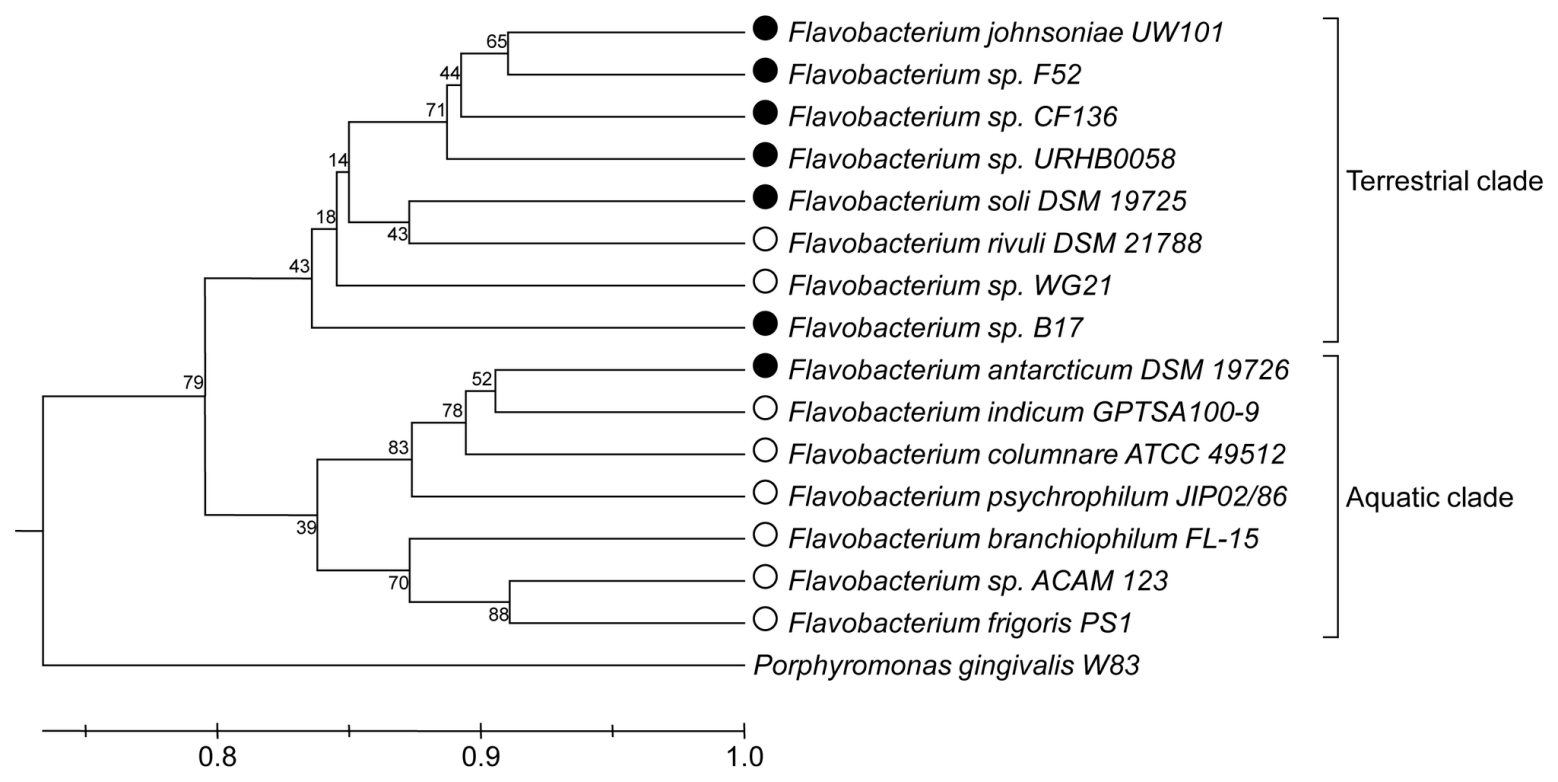
Hierarchical clustering of *Flavobacterium* strains based on functional similarity. *Flavobacterium* strains functionally clustered into terrestrial and aquatic clades. The distribution of genes across SEED subsystems was normalized to the total number of genes in a particular genome. Hierarchical clustering was performed using the Bray-Curtis distance matrix. Bootstrap values are shown next to the branch nodes. Open and black circles represent aquatic and terrestrial isolates, respectively.

Analysis of protein distribution of *Flavobacterium* strains based on functional (SEED) subgroups generated two distinct clusters: a mostly terrestrial clade consisting of temperate soil and plant-associated flavobacteria, and a predominantly aquatic clade (Figure 2). The exceptions to this division were the aquatic strains *Flavobacterium* sp. WG21 and *F* *. rivuli* , who functionally clustered with terrestrial isolates; and the soil isolates *F* *. antarticum* and *F* *. soli* , which were associated with the aquatic clade (Figure 2).

**Figure 3:**
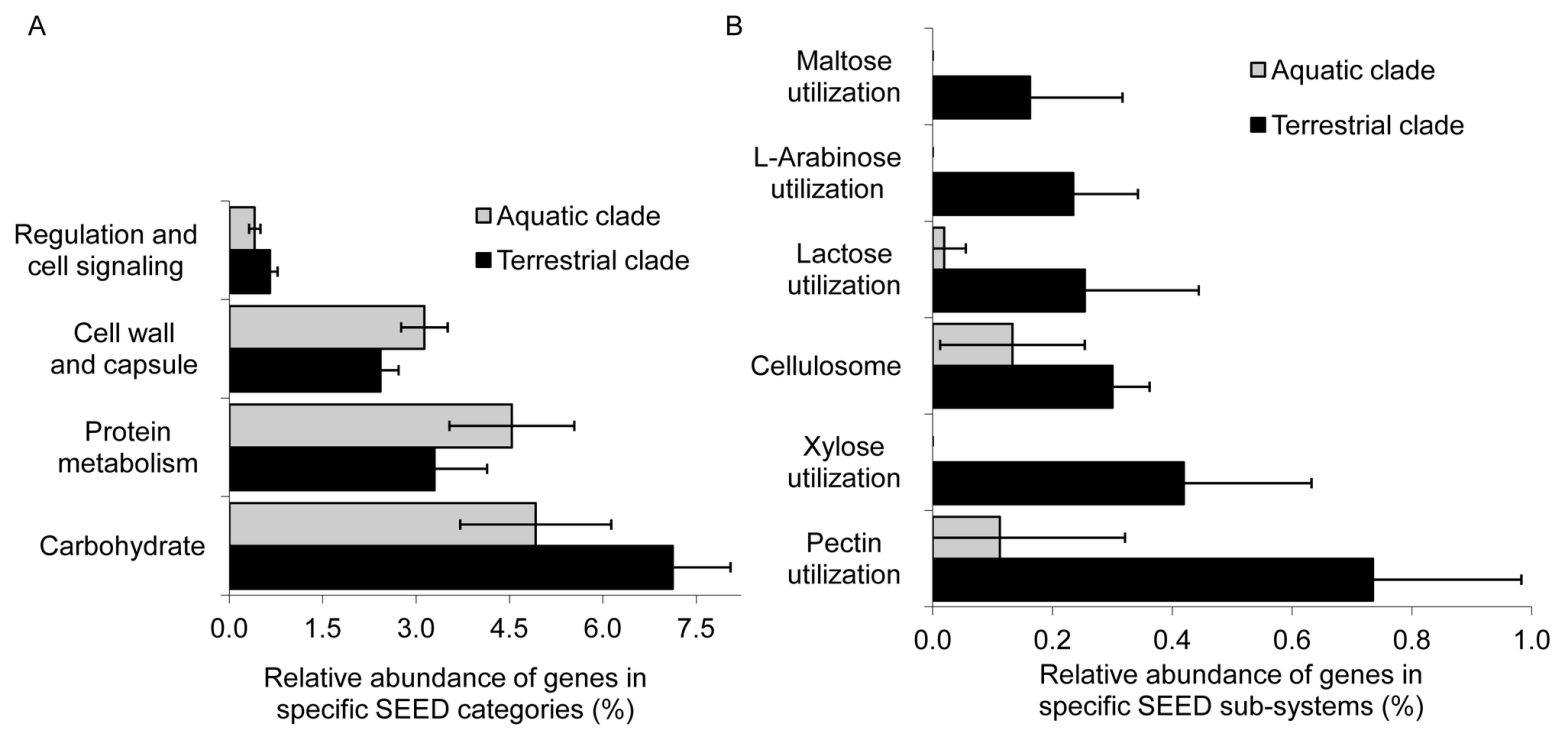
Statistically significant differences in distribution of SEED subsystems between aquatic and terrestrial clades. (A) Major SEED categories, and (B) carbohydrate sub-systems, showing significant differences between aquatic and terrestrial clades. The distribution of genes across SEED categories and subsystems were normalized to the total number of genes in a particular genome and Student T-test (*P*<0.05) was applied to test statistical significance.

In order to identify proteins potentially associated with plant-flavobacterial interactions, we focused on SEED categories that were substantially over-represented among members of the terrestrial clade. These included genes associated with cell signaling pathways and genes encoding for carbohydrate metabolism (Figure 3A) as well as genes associated with Ton and Tol transport systems; metal and drug resistance; and denitrification. Additionally, our analysis revealed that SEED sub-systems associated with metabolism of plant related carbohydrates such as xylose, arabinose and pectin were substantially over-represented in the terrestrial clade (Figure 3B). On the other hand, the aquatic clade was characterized by higher representation of genes associated with cell wall and capsule synthesis, protein metabolism (Figure 3A) and genes associated with phosphate transport.

### *Flavobacterium* *core* and niche-specific genomes

Flavobacterial core and aquatic and terrestrial niche-specific genomes were generated using the methodology described in the materials and methods section, with orthologous genes being characterized as sharing at least 60% similarity in amino acid sequence and less than 30% difference in protein length. The flavobacterial core genome consisted of only 227 predicted proteins, representing 7.3±1.3 and 5.2±0.5% of all predicted proteins across aquatic and terrestrial flavobacterial clades, respectively. In contrast, 798 orthologous ORFs were terrestrial niche-specific, while only 32 unique ORFs were aquatic niche-specific (Table S2). Interestingly, 56% (18/32) of the aquatic niche-specific and 39% (315/798) of the terrestrial niche-specific ORFs encoded for hypothetical proteins, whereas only 4% (9/227) of the flavobacterial core genome ORFs were hypothetical (Table S2). The *Flavobacterium* core genome was primarily associated with basic housekeeping functions such as nucleic acid metabolism, protein synthesis, glycolysis related proteins and amino acid metabolism, with underrepresentation of genes encoding bacterial cell wall modification and transport (Figure S4A). Conversely, the terrestrial-specific genome was significantly enriched with genes related to hydrolysis of glycosidic bonds, nitrogen metabolism and cell signaling and communication, with underrepresentation of CDSs involved in protein biosynthesis and general cellular metabolic processes (Figure S4B).

### Carbohydrate and peptide utilization

**Figure 4:**
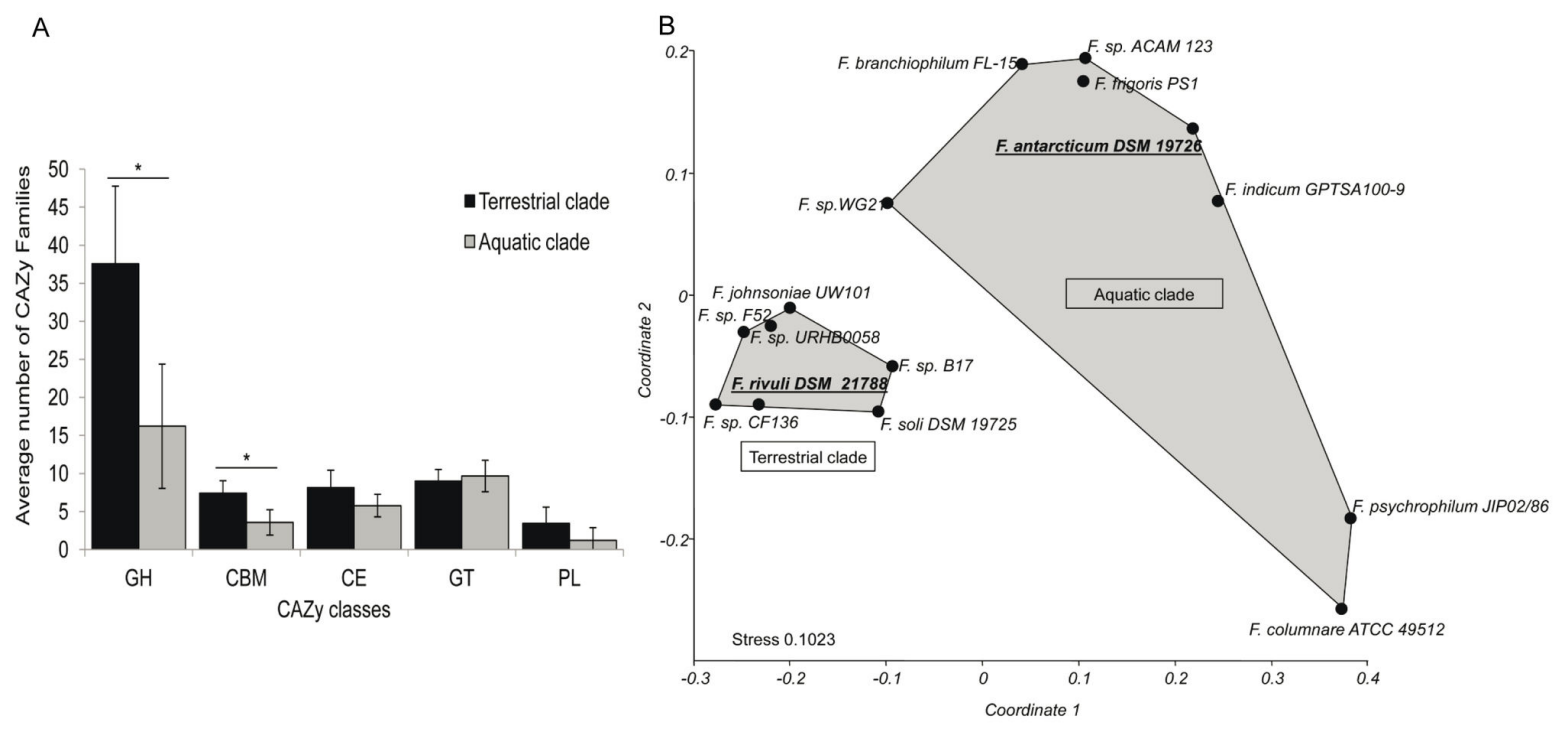
Distribution of carbohydrate-utilizing enzymes. (A) Distribution of CAZy enzyme classes in the terrestrial and aquatic flavobacterial clades; and (B) Distribution of *Flavobacterium* strain glycoside hydrolases visualized using nMDS of a Bray-Curtis distance matrix. GH- glycoside hydrolases; CE-carbohydrate esterase; PL-polysaccharide lyases; CBM- carbohydrate binding modules; GT- glycosyl transferases. Asterisks indicate statistically significant differences (*P* < 0.05) between clades.

The SEED analyses described above, stimulated more comprehensive investigation of genes involved in carbohydrate metabolism. Abundance and diversity of Carbohydrate-Active EnZyme database (CAZy)-characterized enzymes were found to be significantly higher in the terrestrial clade genomes than in the aquatic clade genomes (Figure 4A, Table S3). Specifically, the terrestrial clade genomes contained significantly higher variation and abundance of glycoside hydrolase (GH) and Carbohydrate-Binding Module (CBM) families (Figure 4A). Bray-Curtis based nMDS ordination of GH families clearly indicated environmental niche differentiation of bacteria belonging to the two clades (Figure 4B). Two exceptions were *F* *. antarticum* which was associated with the aquatic clade, and the stream isolate *F* *. rivuli* , which was strongly affiliated with the terrestrial clade (Figure 4B). The terrestrial niche-specific genome contained unique CAZy enzymes involved in metabolism of glucans, arabinose and rhamnogalacturonan that are exclusively associated with terrestrial plant hemicellulose (Table 2, Table S3).

**Table 2:** Terrestrial clade-unique CAZy domains.

	Rhamnogalacturonan			Xylan				Arabinose			Mannose		Fucose	Pectin	Chitin	
Strain	GH 28	GH 78	GH 106	GH 10	GH 115	CE 2	CBM 6	GH 27	GH 43	GH 51	GH 1	GH 130	GH 95	PL 1	GH 18	GH 89
* Flavobacterium johnsoniae *	7	3	2	3	1	1	4	2	10	2	1	5	2	7	4	1
* Flavobacteriumsp. F52*	6	3	2	1	1	1	6	2	13	2	2	5	2	6	3	1
* Flavobacteriumsp. CF136*	10	4	2	3	1	1	3	4	25	6	1	4	5	11	3	1
* Flavobacteriumsp. URHB0058*	11	3	2	1	3	1	3	1	10	3	1	4	2	5	3	1
* Flavobacterium rivuli *	4	1	2	0	0	1	1	2	13	3	2	2	3	1	0	0

* Flavobacteriumsp. B17*	0	0	0	1	2	0	4	1	8	1	0	1	1	0	1	1
* Flavobacterium soli *	1	0	0	0	3	1	2	1	7	0	1	2	0	1	0	0
* Flavobacteriumsp. WG21*	3	0	0	0	0	0	1	0	1	0	1	0	0	2	3	0

* Flavobacterium antarcticum *	0	0	0	0	0	0	1	0	0	0	0	0	0	0	0	0
* Flavobacterium branchiophilum *	1	0	0	0	0	0	0	0	0	0	0	0	0	0	0	0
* Flavobacterium columnare *	0	0	0	0	0	0	0	0	0	0	0	0	0	0	0	0
* Flavobacterium indicum *	0	0	0	0	0	0	0	0	0	0	0	0	0	0	0	0
* Flavobacterium psychrophilum *	0	0	0	0	0	0	0	0	0	0	0	0	0	0	0	0
* Flavobacterium frigoris PS1*	0	0	0	0	0	0	0	0	0	0	0	0	0	0	0	0
* Flavobacteriumsp. ACAM 123*	0	0	0	0	0	0	0	0	0	0	0	2	0	0	0	0

GH- glycoside hydrolases; CE-carbohydrate esterase; PL-polysaccharide lyases; CBM- carbohydrate binding modules; GT- glycosyl transferases.

**Figure 5:**
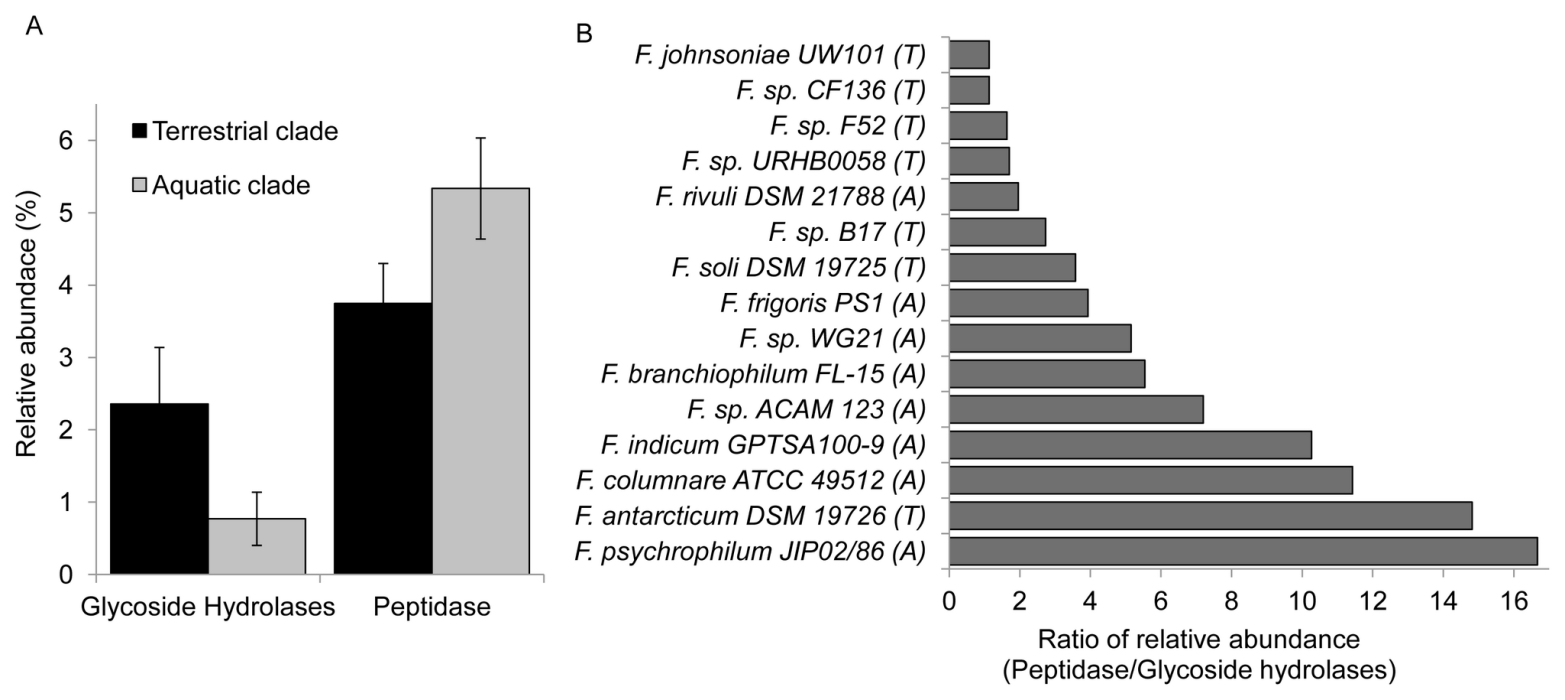
Relative abundances of hydrolytic enzymes. (A) Relative abundances of peptidases and glycoside hydrolases in terrestrial and aquatic flavobacterial clades; (B) Peptidase/GH ratios of *Flavobacterium* strains analyzed in this study; letters A and T in the parentheses indicates aquatic or terrestrial origin of strains, respectively. Functional predicted peptidases and glycoside hydrolases were normalized to the total number of genes in a particular genome.

While the terrestrial clade strains had significantly higher relative abundances of genes encoding GH enzymes, the aquatic strains appeared to be more tailored for peptide utilization (Figure 5A), supporting previous findings by Fernández-Gómez et al, (2013). This is strongly visualized by the peptidase/GH ratio, which averaged 9.7±4.7 in the aquatic clade *vs.* 1.7±0.7 in the terrestrial clade. The fish pathogen *F* *. psychrophilum* had the highest ratio (16.7), whereas the soil and rhizosphere isolates *F* *. johnsoniae* *and* *Flavobacterium* sp. CF136 had the lowest ratios (1.1) (Figure 5B).

## Discussion

A major challenge of microbial ecology is to determine the functional capacity of dominant organisms in specific environmental niches. *Flavobacteria* are ubiquitous in a wide array of terrestrial and aquatic environments where they are believed to play a pivotal role in mineralization of poorly degradable macromolecules and thus serve as carbon flux regulators in these ecosystems [39]. A myriad of recent molecular and culture-based data has strongly indicated that flavobacteria are highly enriched in the phyllosphere and rhizoplane of an array of plants, where they may represent over 20% of the bacterial community [2-4,6-8]; suggesting that they may play a pivotal role in these environments. However, we know practically nothing about the genetic characteristics that are responsible for their capacity to thrive in these environments. In order to identify specific functional properties that are required for flavobacterial adaptation to plant interfaces, we compared the recently sequenced root-associated *Flavobacterium* sp. F52 genome [15] to 14 additional *Flavobacterium* genomes from a wide range of terrestrial and aquatic ecosystems (Table 1). Phylogenetic and functional gene-based analyses of flavobacterial genomes generated two clusters comprised primarily of aquatic and terrestrial strains, respectively (Figure 2).

As discussed above, comparative analysis of the two clades revealed significant differences in the allocation of carbohydrate-utilizing genes (Figure 3A), and highly significant overrepresentation of genes that degrade plant related carbohydrates such as xylose, arabinose and pectin in the terrestrial clade (Figure 3B). Furthermore, the terrestrial clade-affiliated flavobacteria harbored a wide array of unique CAZy enzymes involved in the metabolism of glucans containing arabinose (GH27 and GH43) and rhamnogalacturonan (GH78 and GH106) that are exclusively associated with terrestrial plant hemicellulose (Table 2) [40,41]. These GH’s were absent in all of the analyzed aquatic strains (exceptions discussed below), potentially indicating co-evolution of terrestrial flavobacterial plant-associated glycoglucanases with terrestrial plants. It was previously suggested that the scope of GH’s in a particular microorganism and their capacity to utilize particular carbon sources, is dictated by the ecological niche that they inhabit [42]. For instance, *Bacteroidetes* from phytoplankton-rich marine environments have higher potential for degradation of sulfated algae-derived polysaccharides like fucoidan and (xylo)mannan than their relatives from the oligotrophic areas, whose metabolic potential appeared to be more geared towards peptide utilization and light harvesting as described below [43].

Our results strongly support this notion, and we postulate that GHs may be accurate genomic predictors for identification of plant-associated flavobacteria. For example, SEED-defined functional cluster analyses (Figure 2), as well as detailed analysis of the flavobacterial GH distributions (Figure 4B, Table 2, Table S3), specifically designated the stream isolate *F* *. rivuli* [44] as a member of the terrestrial clade, contrary to its apparent aquatic origin. This was supported by the presence of glycoside hydrolases GH78 and GH106 (Table 2, Table S3), which are responsible for rhamnogalacturonan cleavage which is incorporated mainly into cell walls of all land plants [40,41]. The notion that *F* *. rivuli* is in essence soil-derived is supported by a previous microbial fingerprinting-based study that analyzed stream and soil flavobacterial communities from in and around the stream from which *F* *. rivuli* was isolated [45]. Based on these analyses the authors inferred that the flavobacterial stream community, originated from soil washout [45]. Conversely, functional and GH clustering (Figure 2, Figure 4B) placed *F* *. antarcticum* , originally isolated from an Antarctic soil [46], within the aquatic clade, which is supported by the absence of GH78 and GH106 in this bacterium (Table 2, Table S3). We hypothesize that this stems from the absence of terrestrial plants in the isolated environment, emphasizing the notion that terrestrial plant glycans dictate the arsenal of GH’s in the terrestrial flavobacterial clade.

Almost all *Flavobacterium* GH’s are clustered together with TonB-dependent receptors and transducers in small genomic islands, suggesting an integrated regulation of adhesion and degradation of polymers [47]. There is significant evidence that extracellular chitinase genes, exclusive to the terrestrial flavobacterial genomes are the product of horizontal gene transfer, most likely from *Firmicutes* phylum (data not shown). In contrast, based on BLASTP (http://blast.ncbi.nlm.nih.gov) similarities, most of the soil-unique GH’s are highly conserved within the *Bacteroidetes* phylum indicating that these genes were lost by the aquatic strains. Interestingly, the cluster affiliation of the soil strain *F* *. soli* , isolated from a recently-formed 0.2 km^2^ volcanic island off the coast of South Korea [48], changed depending on the specific genomic element being analyzed. For instance functional gene-based clustering determined that it was affiliated with the aquatic clade (Figure 2), whereas GH-based nMDS analysis placed it strongly within the terrestrial clade (Figure 4B).

In general, the genomes of the terrestrial clade were approximately 40 percent larger than the aquatic clade genomes (Table 1, Figure S1), with the exceptions discussed above behaving according to their corresponding clades for this parameter as well. Bacteria genome size can be strongly shaped by environmental complexity [49]. For instance free-living bacteria that have more heterogeneous lifestyles generally harbor large genomes, especially in complex environments with strong physico-chemical gradients [50]. Conversely, facultative pathogens generally contain smaller genomes, while, obligatory pathogens are readily characterized by extremely reduced genome size [51]. Whole genome comparison of the flavobacteria genomes coincides with this general rule, with an apparent correlation between genome size and environmental heterogeneity. The evidently free living soil isolate *F* *. johnsoniae* had the largest genome size followed by the rhizosphere strain *Flavobacterium* sp. F52, whereas the poplar root and rice endophytes *Flavobacterium* sp. CF136 and *Flavobacterium* sp. B17, respectively, had the smallest genomes among the terrestrial plant-associated flavobacteria analyzed in this study. The same tendency was observed within the aquatic clade. The free living strain *Flavobacterium* sp. GW21 had the largest genome, whereas the fish pathogens harbored much smaller genomes (Table 1). Temperate soils are characterized by a highly complex multi-dimensional architecture that is often associated with strong spatial and temporal gradients in a wide array of biotic and a-biotic parameters including: plant biomass water potential, oxygen, nutrients, temperature and pH [52,53]. Therefore, it is perhaps not surprising to find larger genomes with overrepresentation of sensory and signal transduction proteins in the terrestrial clade niche-specific genome (Figure 3A, Figure S4, Table S2).

*Flavobacteria*, the most abundant class of *Bacteroidetes* in marine environments [54], are assumed to specialize in the degradation of polymers and particulate organic matter (POM) [5,54-57]. Amino acids, peptides and proteins appear to be important components of POM, and these components account for up to 15% of total biologically available nitrogen in the ocean [58]. Therefore, given the general limitation of nitrogen in ocean environments, the ability to utilize peptide-incorporated POMs may be beneficial for bacteria propagation. *Flavobacteria* adaptation to oligotrophic condition appears to be primarily associated with enhanced peptide utilization and the capacity to utilize alternative energy sources such as light, which is harvested with proteorhodopsin [59]. Moreover, the synthesis and expression of proteolytic and peptidoglycan degrading enzymes are much higher than in mesotrophic or eutrophic areas [43]. This is demonstrated by the high number of peptidases found in genome sequences of the Proteorhodopsin-containing marine *Flavobacterium* Dokdonia sp. MED134 and *Polaribacter* sp. MED152 isolated from oligotrophic Mediterranean sea [60,61].

We believe that the high peptidase/GH ratio (>6) detected in the aquatic clade genomes, implies that this clade is primarily associated with protein-amino acid metabolism, in contrast to the terrestrial strains that had a much lower peptidase/GH ratio (~1.5), who seem to depend primarily on plant related polysaccharide degradation. This is supported by our analysis (Figure 5B) and by a recently published comparative genomic studies of *Bacteroidetes*, which indicated that the peptidase/GH ratio in the genomes of aquatic *Bacteroidetes* were above six, while the soil species ratio was approximately one [43,47,62]. The exceptions to this rule are *F* *. antariticum* , whose peptidase/GH ratio (15) (Figure 5B) was far higher than other soil isolates and *F* *. rivuli* , whose peptidase/GH ratio (1.9) was much more similar to terrestrial strains. The *F* *. antariticum* aquatic-like peptidase/GH ratio is also supported by it smaller genome size (3.08 Mbp), and we hypothesize that its aquatic-like attributes are most likely due to the lack of terrestrial plant material in the Antarctic soils and from the fact that it potentially originated from the surrounding seawater. Conversely, the terrestrial-like peptidase/GH ratio of *F* *. rivuli* , its larger genome size (4.49 Mbp) and arsenal of genes encoding plant glycan metabolizing enzymes, strongly indicate that it is of terrestrial origin. The intermediate peptidase/GH ratio (3.6) and genome size (4.0 Mbp) of *F* *. soli* suggest that it is adapted to aquatic/terrestrial interfaces, or perhaps represents a transitional stage between aquatic and terrestrial lifestyles. This is potentially supported by the fact that it was isolated from a new volcanic island of the coast of Korea, which contains almost no soil and very sparse vegetation. Large-scale genome screening of flavobacterial strains from a myriad of terrestrial and aquatic environments is required to enhance our understanding of these relationships and our comprehension of how flavobacterial genomes evolved to adapt to different environments.

## Supporting Information

Figure S1 **Genome size of aquatic and terrestrial flavobacterial clades.**
(TIF)

Figure S2 **Phylogenetic relationships of 16S rRNA genes in *Flavobacterium* strains.** Maximum Likelihood analysis based on concatenated alignments of 16S rRNA genes. Bootstrap values are shown next to the branch nodes. Open and black circles represent aquatic and terrestrial isolates, respectively.
(TIF)

Figure S3 **Phylogenetic relationships of core genes in *Flavobacterium* strains.** Maximum Likelihood method is based on Hal-software-generated 15 concatenated core genome polypeptides with 100 bootstrap replicates. The percentage of trees in which the associated taxa clustered together is shown next to the branches. Open and black circles represent aquatic and terrestrial isolates, respectively.
(TIF)

Figure S4 **Over- and under-represented gene ontologies (GO’s) in the *Flavobacterium* core and niche-specific genomes.** (A) *Flavobacterium* core genome and (B) terrestrial clade niche-specific genome. Values over one (dotted line) represent GO’s terms that are overrepresented, where values under one are underrepresented. Enrichment of GO’s terms was tested using a one-tail Fisher’s exact test, between *Flavobacterium* core and niche-specific genomes and the whole protein set of *F* *. johnsoniae* .
(TIF)

Table S1 **General characteristics of the pepper root-associated flavobacterial isolates.** Phenotypic and growth characteristics were measured as follow: Flexirubin: *Flavobacterium* spp. were grown on CYE-agar for 24 hours and the exposure to 50 µl of 10 N KOH followed by exposure to 42 µl 12 N HCl. Flexirubin-positive cells were yellow at neutral pH and red under alkaline conditions. Gliding motility: *Flavobacterium* spp. were grown on PY2 agar medium (2 g of peptone, 0.5 g yeast extract, 5 g agar and DDW up to 1 litter) at 25°C. Colony spreading was observed by binocular 24-48 hours after initial platting. Hydrogen cyanide (HCN) production: *Flavobacterium* spp. were grown on PY2 agar medium supplemented with glycine (4.4 g per litter) and cyanogenesis was revealed as previously described by 23. Results were read after 4 days of culture at 28°C. A change in filter paper colour from yellow to orange-brown indicated production of HCN (Yellow (1) - limited cyanide production; orange (2) - moderate cyanide production; light brown (3) – relatively high cyanide production; and brown (4) - high cyanide production). Amonia (NH_3_) production was estimated by using Nesller’s reagent according to the manufacturer’s instructions. Generation times of *Flavobacterium* spp. under different conditions were calculated from measured OD_600nm_ at 25°C and 200 rpm. and indicates as follow: (5)- 60-90 min, (4)- 90-120 min, (3)- 120-200 min, (2)- 200-500 min, (1)- 500-800 min, (0)- 800-1200 and N.D for not detectible growth or generation time was longer than 1200 min. Hydrolytic activities were measured based on standard procedures.
(XLSX)

Table S2 **Flavobacterial clade-specific proteins.** Orthologs were defined by identifying unique pairwise reciprocal best hits, with at least 60% similarity in amino acid sequence and less than 30% difference in protein length. To determine the flavobacterial core genome, we used the 15 available genomes. For niche-specific core genome identification we used the completed genomes *F* *. branchiophilum* FL-15, *F* *. psychrophilum* JIP02/86; *F* *. columnare* ATCC 49512; *F* *. indicum* GPTSA100-9; *F* *. johnsoniae* UW101 and two draft genomes *Flavobacterium* sp. F52 and *Flavobacterium* sp. CF136.
(XLSX)

Table S3 **Summary of distribution carbohydrate utilizing enzymes.** Only sequences with similar predictions from dbCAN prediction web server and CAZymes Analysis Toolkit were selected and named according to CAZy classifications. GH- glycoside hydrolases; CE-carbohydrate esterase; PL-polysaccharide lyases; CBM- carbohydrate binding modules; GT- glycosyl transferases.
(XLS)
