# Correction: Comparative Genomic Analysis Indicates that Niche Adaptation of Terrestrial *Flavobacteria* Is Strongly Linked to Plant Glycan Metabolism

**DOI:** 10.1371/annotation/3eafbdc9-374a-447a-852b-7a64503b8ccb

**Published:** 2014-01-06

**Authors:** Max Kolton, Noa Sela, Yigal Elad, Eddie Cytryn

Affiliation number 3 for the first author, Max Kolton, is incorrect. The correct affiliation is: Agroecology and Plant Health Program, The Robert H. Smith Faculty of Agriculture, Food and Environment, the Hebrew University of Jerusalem, Rehovot, Israel. 

