# Supplementary figures and images for: Comparative Genomic Analysis Indicates that Niche Adaptation of Terrestrial Flavobacteria Is Strongly Linked to Plant Glycan Metabolism
Source: PLoS One. 2013 Sep 26;8(9):e76704. doi: 10.1371/journal.pone.0076704 (PMC3784431; doi:10.1371/journal.pone.0076704)

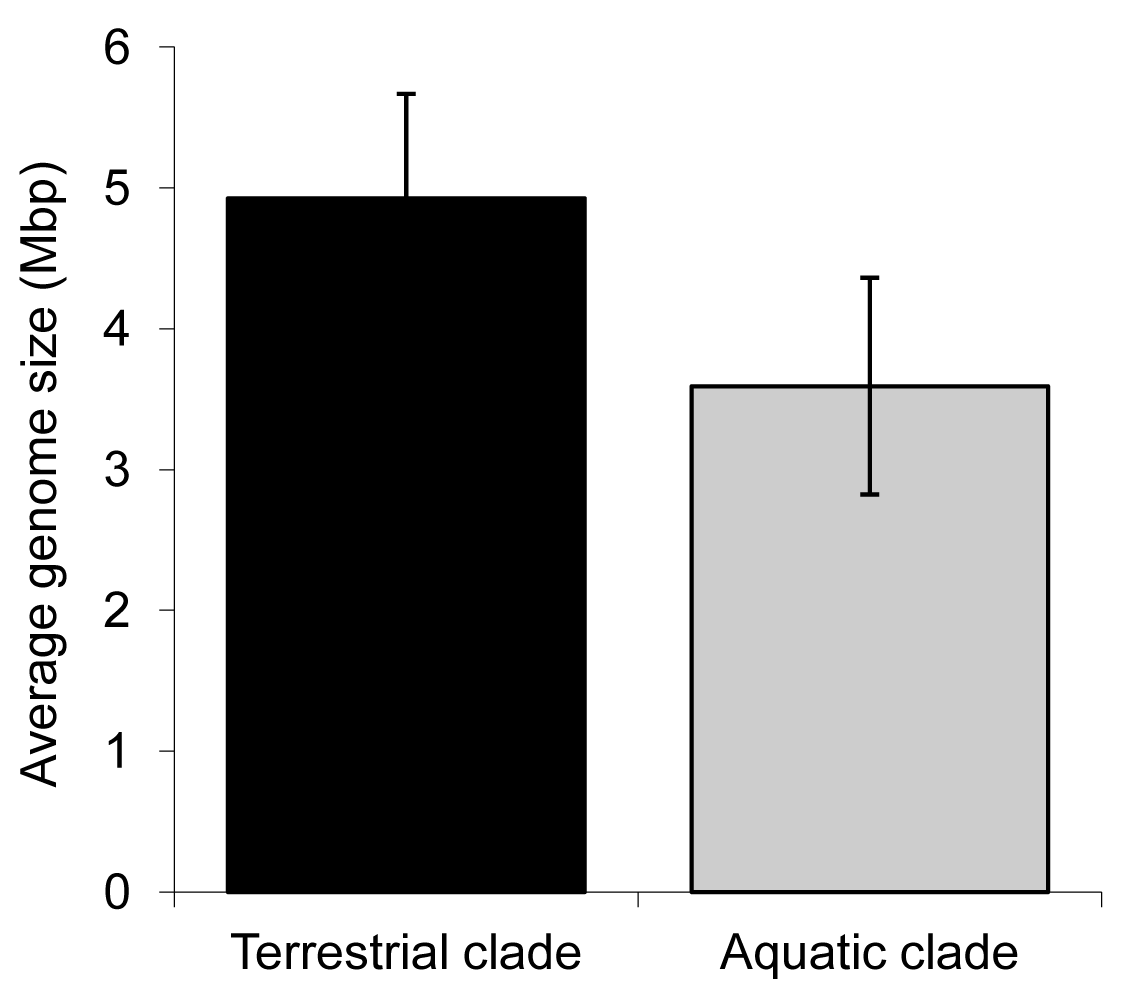

Supplement: Figure S1 — Genome size of aquatic and terrestrial flavobacterial clades. (TIF) [file pone.0076704.s001.tif]

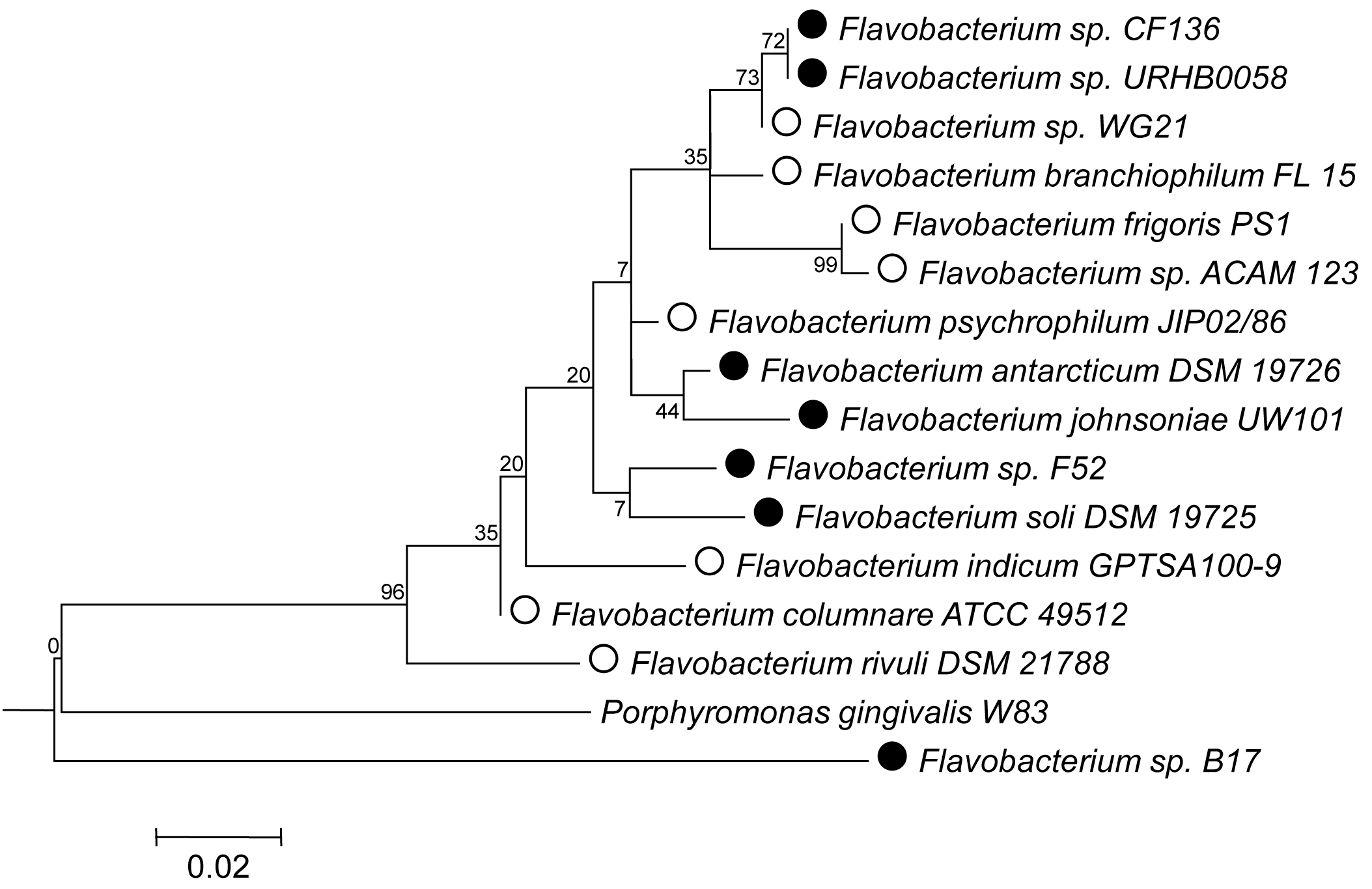

Supplement: Figure S2 — Phylogenetic relationships of 16S rRNA genes in Flavobacterium strains. Maximum Likelihood analysis based on concatenated alignments of 16S rRNA genes. Bootstrap values are shown next to the branch nodes. Open and black circles represent aquatic and terrestrial isolates, respectively. (TIF) [file pone.0076704.s002.tif]

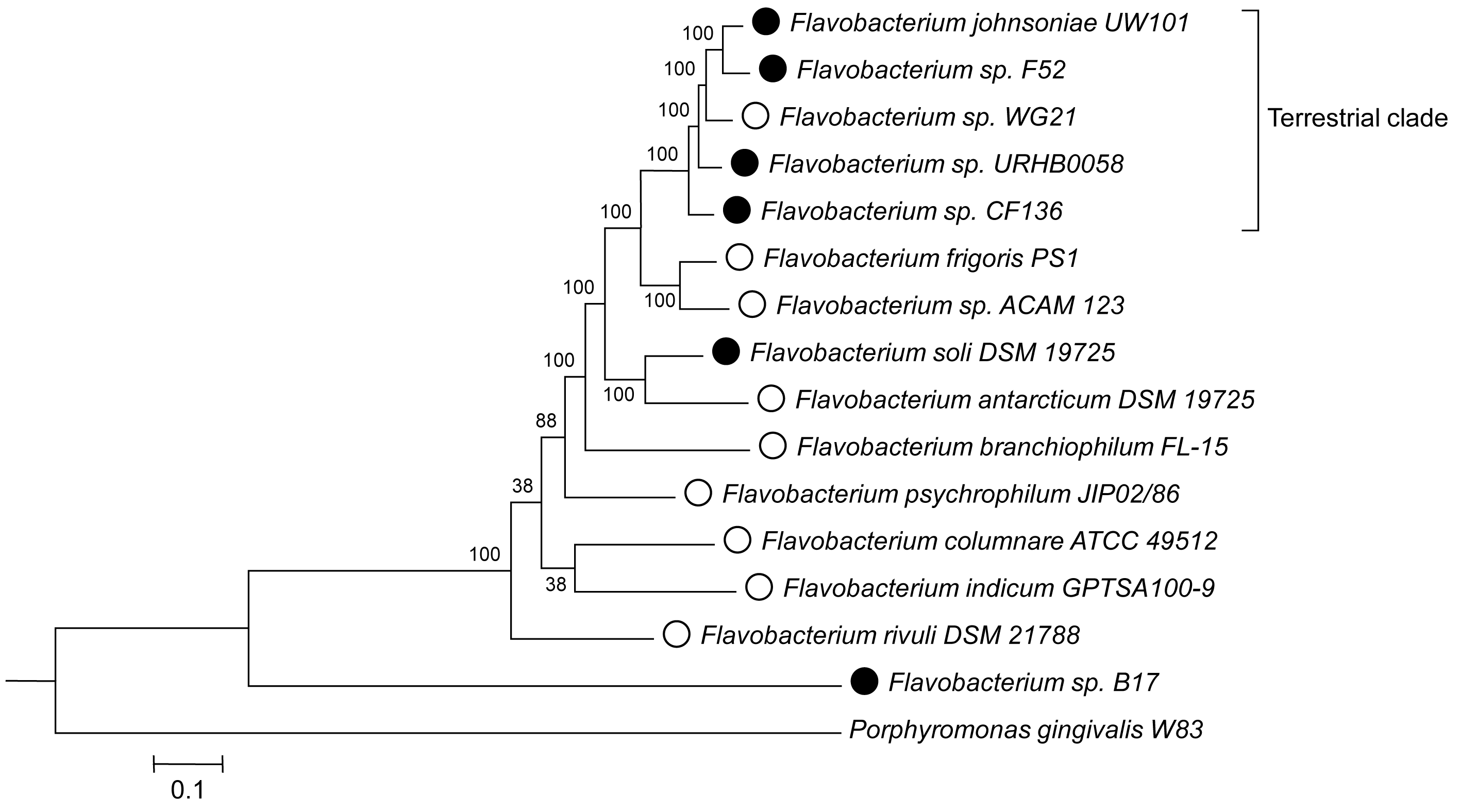

Supplement: Figure S3 — Phylogenetic relationships of core genes in Flavobacterium strains. Maximum Likelihood method is based on Hal-software-generated 15 concatenated core genome polypeptides with 100 bootstrap replicates. The percentage of trees in which the associated taxa clustered together is shown next to the branches. Open and black circles represent aquatic and terrestrial isolates, respectively. (TIF) [file pone.0076704.s003.tif]

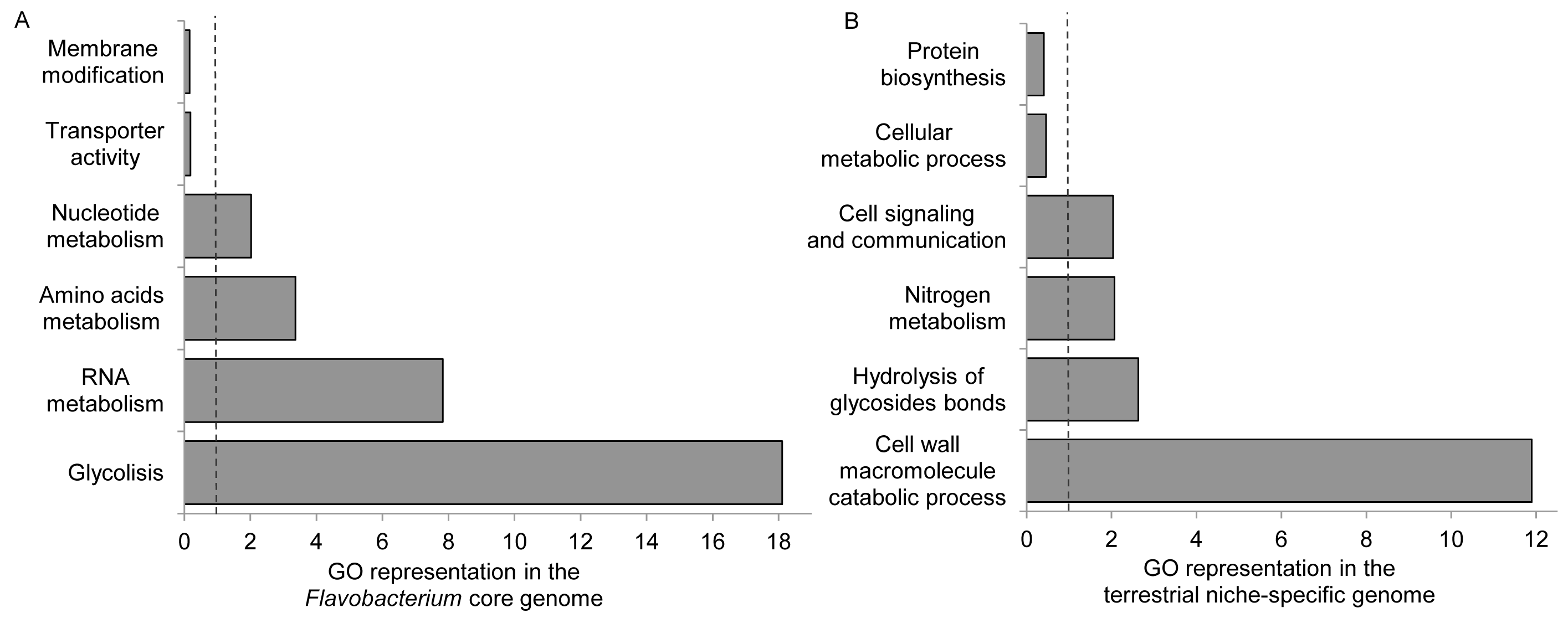

Supplement: Figure S4 — Over- and under-represented gene ontologies (GO’s) in the Flavobacterium core and niche-specific genomes. (A) Flavobacterium core genome and (B) terrestrial clade niche-specific genome. Values over one (dotted line) represent GO’s terms that are overrepresented, where values under one are underrepresented. Enrichment of GO’s terms was tested using a one-tail Fisher’s exact test, between Flavobacterium core and niche-specific genomes and the whole protein set of F . johnsoniae . (TIF) [file pone.0076704.s004.tif]
